# Balancing of Motor Armature Based on LSTM-ZPF Signal Processing

**DOI:** 10.3390/s22239043

**Published:** 2022-11-22

**Authors:** Ruiwen Dong, Mengxuan Li, Ao Sun, Zhenrong Lu, Dong Jiang, Weiyu Chen

**Affiliations:** 1 School of Mechanical and Electronic Engineering, Nanjing Forestry University, Nanjing 210037, China; 2 Institute of Aerospace Machinery and Dynamics, Southeast University, Nanjing 211189, China

**Keywords:** LSTM, ZPF, motor armature, unbalance signal, dynamic balancing

## Abstract

Signal processing is important in the balancing of the motor armature, where the balancing accuracy depends on the extraction of the signal amplitude and phase from the raw vibration signal. In this study, a motor armature dynamic balancing method based on the long short-term memory network (LSTM) and zero-phase filter (ZPF) is proposed. This method mainly focuses on the extraction accuracy of amplitude and phase from unbalanced signals of the motor armature. The ZPF is used to accurately extract the phase, while the LSTM network is trained to extract the amplitude. The proposed method combines the advantages of both methods, whereby the problems of phase shift and amplitude loss when used alone are solved, and the motor armature unbalance signal is accurately obtained. The unbalanced mass and phase are calculated using the influence coefficient method. The effectiveness of the proposed method is proven using the simulated motor armature vibration signal, and an experimental investigation is undertaken to verify the dynamic balancing method. Two amplitude evaluation metrics and three phase evaluation metrics are proposed to judge the extraction accuracy of the amplitude and phase, whereas amplitude and frequency spectrum analysis are used to judge the dynamic balancing results. The results illustrate that the proposed method has higher dynamic balancing accuracy. Moreover, it has better extraction accuracy for the amplitude and phase of unbalanced signals compared with other methods, and it has good anti-noise performance. The determination coefficient of the amplitude is 0.9999, and the average absolute error of the phase is 2.4°. The proposed method considers both fidelity and denoising, which ensuring the accuracy of armature dynamic balancing.

## 1. Introduction

Manufacturing assembly errors and inhomogeneity in materials lead to mass unbalance of the motor armature, which leads to vibration and noise during the service of the motor, affecting the performance, efficiency, and service life of the motor, and even causing safety accidents [1,2]. Dynamic balancing of the armature is crucial in motor manufacturing [3]. Most of the motor armatures can be treated as rigid rotors, where their operating speed is below the first-order critical speed, which means the effect of the operational deflection can be ignored [4,5]. The unbalanced signal at this point can be viewed as a sinusoidal function [6]. Rigid rotor balancing generally uses the double-sided influence coefficient method to calculate the unbalanced mass and phase [7,8], and the accuracy of its correction is closely related to the results of the rotor unbalance signal extraction [9]. As the actual signal acquisition is affected by the test instrument, test environment, and human factors, noise also exists in unbalance signal [10]. Therefore, the effect of vibration signal denoising and filtering has a great impact on the accuracy of dynamic balance correction.

Classical digital filters are often used for vibration signal filtering [11,12]. In addition, some new methods have been proposed, such as time–frequency analysis using the bispectrum and the autocovariance of stray flux signals [13], variable-step fourth-order Runge–Kutta iteration [14], discrete wavelet transform [15], zoom synchronous transformation (ZST) and tacholess sequential tracking [16], and balancing without trial weights based on the dynamic similitude scale model [17]. However, the above methods either have phase shift or amplitude loss. A solution to eliminate the phase shift is the zero-phase filter (ZPF) [18]; by filtering the input data in both forward and reverse directions, the ZPF has precise zero-phase distortion [19,20,21,22]. Although the ZPF can accurately extract the phase, there is a loss in signal amplitude extraction, and it is difficult to give consideration to both fidelity and denoising.

In recent years, neural networks have been widely used in signal processing and denoising, benefiting from the powerful automatic feature extraction ability and massive data processing capability of deep learning. Saeed Anwar [23] proposed a single-stage blind real-image denoising network (RIDNet) using a modular structure based on a convolutional neural network (CNN). Meng Chang [24] proposed a new adaptive denoising network (SADNet) that can effectively remove blind noise from single images. Zejin Wang [25] proposed a self-supervised denoising method that can overcome the information loss caused by the blind point-driven denoising method. All the above methods are based on two-dimensional image noise reduction. On the other hand, most one-dimensional data signal processing methods are based on long short-term memory networks (LSTM). In general, LSTM is considered to be one of the state-of-the-art methods for dealing with time series prediction problems [26,27,28,29]. However, most of the signal processing based on LSTM involves analyzing and predicting its future time-domain signal in a signal sequence [30,31,32].

A dynamic balancing method based on the LSTM network and ZPF is proposed in this paper, which improves the signal attenuation and phase shift caused in the filtering. The phase is extracted using the ZPF. The amplitude is extracted using the memory function of the LSTM for the timing sequence. To verify performance, we conducted some experiments in different noise environments and used five evaluation metrics. The results show that the proposed method can produce higher balancing accuracy than other baseline methods.

## 2. Problem Description

**Figure 1:**
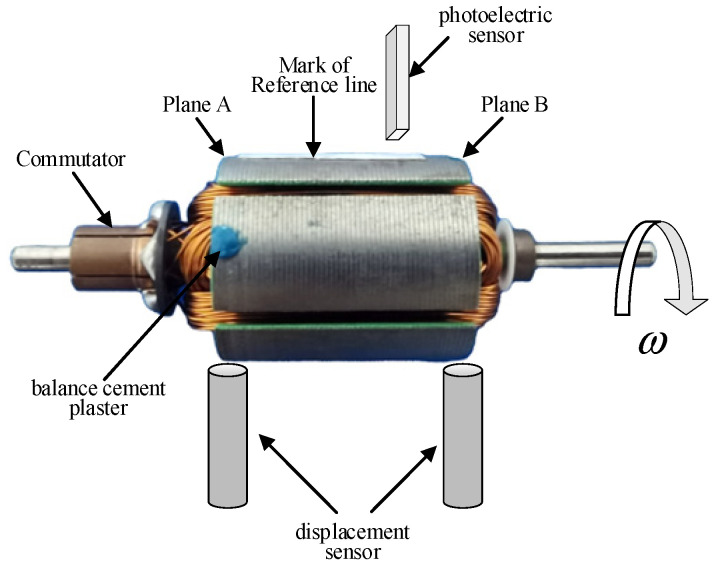
Schematic diagram of armature unbalance signal measurement.

Figure 1 shows the basic configuration of armature dynamic vibration signal acquisition in this paper. The extraction of the vibration signals is achieved using a photoelectric sensor measuring the phase and two displacement sensors measuring the amplitude, which respectively detect the pulse signal of the armature and the vibration signal of the armature under the inertial force. The armature unbalanced mass is calculated on the basis of the measured signal after processing using the influence coefficient method.

The influence coefficient method is to consider the rigid rotor system as a linear system that can be superimposed when balancing the rigid rotor, and the vibration response of each part of the rotor can be linearly superimposed on the two correction planes.

**Figure 2:**
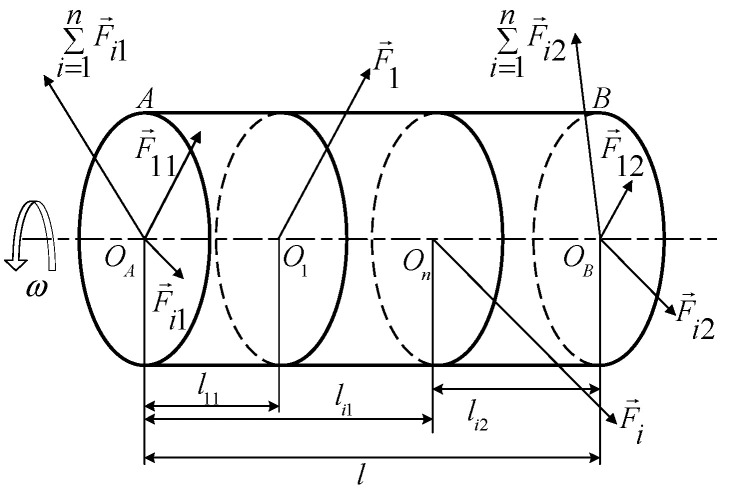
Force analysis of motor armature.

As shown in Figure 2, the impact coefficients of the two correction planes are calculated through the amplitude response of the correction planes. The method is describe below [4,6].

Firstly, the mass m_1_ is added to the correction plane A to obtain two influence coefficients ${\alpha^{\prime }}_{\mathrm{AA}}$ and ${\alpha^{\prime }}_{\mathrm{AB}}$. After removing the weighted mass m_1_, the mass m_2_ is added to the correction plane A to obtain two influence coefficients ${\alpha^{\prime }}_{\mathrm{BA}}$ and ${\alpha^{\prime }}_{\mathrm{BB}}$. The equations are as follows:

(1) $${\alpha^{\prime }}_{\mathrm{AA}}=\left(V_{A1}-V_{A}\right)/m_{1},$$

(2) $${\alpha^{\prime }}_{\mathrm{AB}}=\left(V_{A2}-V_{A}\right)/m_{2},$$

(3) $${\alpha^{\prime }}_{\mathrm{BA}}=\left(V_{B1}-V_{B}\right)/m_{1},$$

(4) $${\alpha^{\prime }}_{\mathrm{BB}}=\left(V_{B2}-V_{B}\right)/m_{2},$$

where $\alpha^{\prime }$ is the influence coefficient, **m** is the mass and phase of the weighted mass block on the rotor, and **V** is the amplitude and amplitude phase for the rotor measurement correction plane.

Therefore, the amount of unbalance on the rotor correction plane corresponds to **M**_A_, **M**_B_, which can be expressed as

(5) $$M_{A}=\frac{{\alpha^{\prime }}_{BB}\times V_{A}}{{\alpha^{\prime }}_{\mathrm{AA}}\times {\alpha^{\prime }}_{BB}-{\alpha^{\prime }}_{\mathrm{AB}}\times {\alpha^{\prime }}_{\mathrm{BA}}}-\frac{{\alpha^{\prime }}_{\mathrm{AB}}\times V_{B}}{{\alpha^{\prime }}_{\mathrm{AA}}\times {\alpha^{\prime }}_{BB}-{\alpha^{\prime }}_{\mathrm{AB}}\times {\alpha^{\prime }}_{\mathrm{BA}}},$$

(6) $$M_{B}=\frac{{\alpha^{\prime }}_{\mathrm{AA}}\times V_{A}}{{\alpha^{\prime }}_{\mathrm{AA}}\times {\alpha^{\prime }}_{BB}-{\alpha^{\prime }}_{\mathrm{AB}}\times {\alpha^{\prime }}_{\mathrm{BA}}}-\frac{{\alpha^{\prime }}_{\mathrm{BA}}\times V_{B}}{{\alpha^{\prime }}_{\mathrm{AA}}\times {\alpha^{\prime }}_{\mathrm{BB}}-{\alpha^{\prime }}_{\mathrm{AB}}\times {\alpha^{\prime }}_{\mathrm{BA}}}.$$

According to Equations (1)–(6), it can be determined that, when **m** is known, a more accurate value of **V** allows a more accurate calculation of unbalance. The reference phase measured by the photoelectric sensor has almost no error; hence, this paper focuses on how to accurately extract the amplitude and phase of the armature unbalance signal from the measured vibration signal containing noise.

## 3. Basic Theory

### 3.1. Long Short-Term Memory (LSTM)

**Figure 3:**
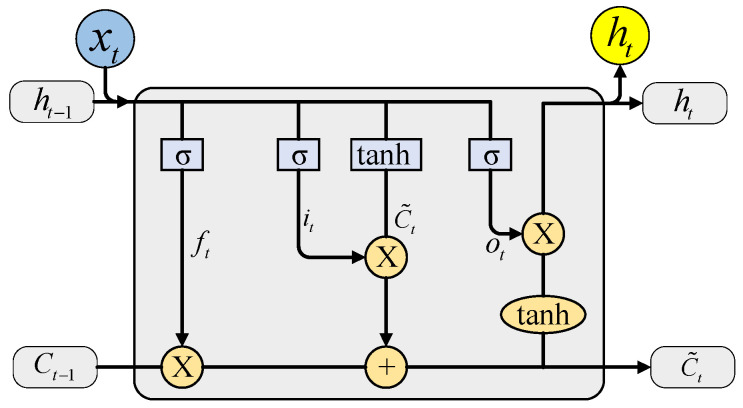
Internal structure of LSTM.

The structure of the LSTM network is shown in Figure 3 [26], which controls the state of information at each moment in the whole neural network through a structure called a “gate” to achieve the learning effect. *C_t_* is the state information of the LSTM unit at time *t*, *f_t_* is the forget gate at time *t*, *i_t_* is the input gate at time *t*, ${\tilde{C}}_{t}$ is the current moment information, *o_t_* is the output gate at time *t*, tanh is the hyperbolic tangent activation function, and *σ* is the sigmoid activation function. The calculation equations are presented below.

Forget layer:

(7) $$f_{t}=\sigma \left(W_{f}\cdot \left[h_{t-1},x_{t}\right]+b_{f}\right).$$

Input layer:

(8) $$i_{t}=\sigma \left(W_{i}\cdot \left[h_{t-1},x_{t}\right]+b_{i}\right).$$

Unit status update layer:

(9) $${\tilde{C}}_{t}=\tanh\left(W_{\tilde{c}}\cdot \left[h_{t-1},x_{t}\right]+b_{\tilde{c}}\right),$$

(10) $$C_{t}=f_{t}\cdot C_{t-1}+i_{t}\cdot {\tilde{C}}_{t}.$$

Output layer:

(11) $$o_{t}=\sigma \left(W_{o}\cdot \left[h_{t-1},x_{t}\right]+b_{o}\right),$$

(12) $$h_{t}=o_{t}\cdot \tanh C_{t}.$$

In the above equations, $W_{f}$, $W_{i}$, $W_{\tilde{c}}$, $W_{o}$ are the weight matrices corresponding to each module, $b_{f}$, $b_{i}$, $b_{\tilde{c}}$, $b_{o}$ are the bias terms, and tanh and σ are defined as

(13) $$\tanh x=\left(e^{x}-e^{-x}\right)/\left(e^{x}+e^{-x}\right),$$

(14) $$\sigma (x)=1/\left(1+e^{-x}\right).$$

Lastly, the output layer is based on equation $h_{t}$ through a fully connected layer to obtain the predicted value $y_{t}$:

(15) $$y_{t}=\sigma \left(W_{y}\cdot h_{t}+b_{y}\right),$$

where $W_{y}$ is the weight matrix, and *b_y_* is the bias term.

LSTM networks can make a more accurate prediction of the later timepoint using the features of the previous timepoint of the time series [33]. The initial position of the previous point is known at this time, which means that the phase of the signal is known. The filtering of the vibration signal can also be seen as a prediction of the later signal time series based on the previous signal time series. Since the starting signal point does not have enough phase characteristics, this can lead to phase information missing in the motor armature unbalance signal processing when using the LSTM network.

### 3.2. Zero-Phase Filter (ZPF)

For one-dimensional time series, the phase shifts of the forward time series and the reverse time series pass through the filter to cancel each other, such that the phase response of the system function is zero; the filtering principle is as follows [21,22]:

(16) $$y_{1}(n)=x(n)\times h(n),$$

(17) $$y_{2}(n)=y_{1}(N-1-n),$$

(18) $$y_{3}(n)=y_{2}(n)\times h(n),$$

(19) $$y(n)=y_{3}(N-1-n),$$

where *x*(*n*) is the original signal sampling sequence, *N* is the length of the signal sequence, *h*(*n*) is the digital filter impact response sequence used, and *y*(*n*) is the reversal sequence of the secondary filtering results.

The filtering process corresponds to the reduced frequency domain expression:

(20) $$Y(e^{jw})=X(e^{jw})|H(e^{jw}){|}^{2},$$

where *ω* is the angular frequency. *X*(*e^jw^*), *Y*(*e^jw^*), and *H*(*e^jw^*) are the discrete Fourier transforms of *x*(*n*), *y*(*n*), and *h*(*n*), respectively.

As can be seen from the equation, there is only a magnitude gain relationship between the output *Y*(*e^jw^*) and the input *H*(*e^jw^*); furthermore, there is no phase shift in the full frequency band. The size of the transition band constructed by the passband and the stopband is inversely proportional to the goodness of the cutoff characteristics. Specifically, a larger transition bandwidth will not allow the filter to adequately suppress the near-frequency interference;, whereas a smaller transition band will cause the actual unbalanced signal of the armature to attenuate and will produce the Gibbs phenomenon, all of which will have an impact on the amplitude phase extraction of the actual unbalanced signal of the armature [34].

## 4. The Proposed Method

**Figure 4:**
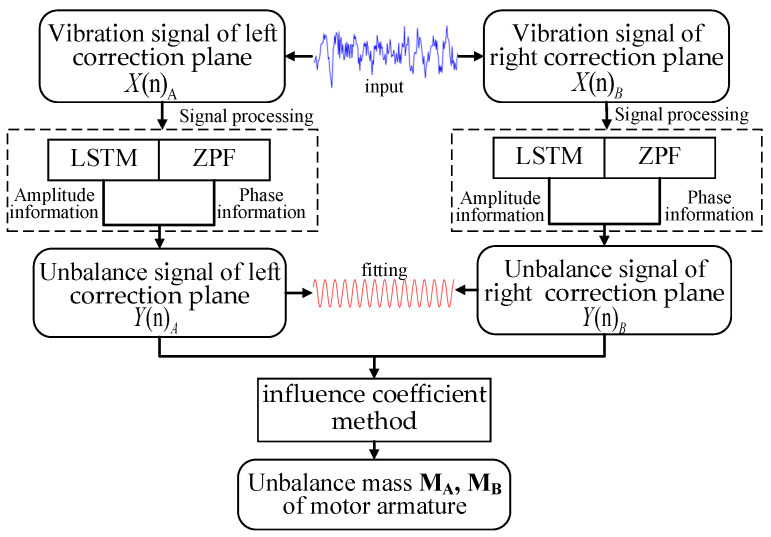
Flowchart of the dynamic balancing method based on LSTM-ZPF.

The proposed LSTM-ZPF method is shown in Figure 4. It mainly consists of two steps: signal processing and dynamic balancing calculation. More details are described in this section.

### 4.1. Principle

The left and right correction plane vibration signals *X*(*n*)*_A_* and *X*(*n*)*_B_* of the motor armature are input into the signal processing section. The amplitude information and phase information are respectively extracted using the LSTM neural network and ZPF. The desired motor armature unbalance signals *Y*(*n*)*_A_* and *Y*(*n*)*_B_* are obtained by fitting. Lastly, the unbalanced mass and phase are calculated using the influence coefficient method.

**Figure 5:**
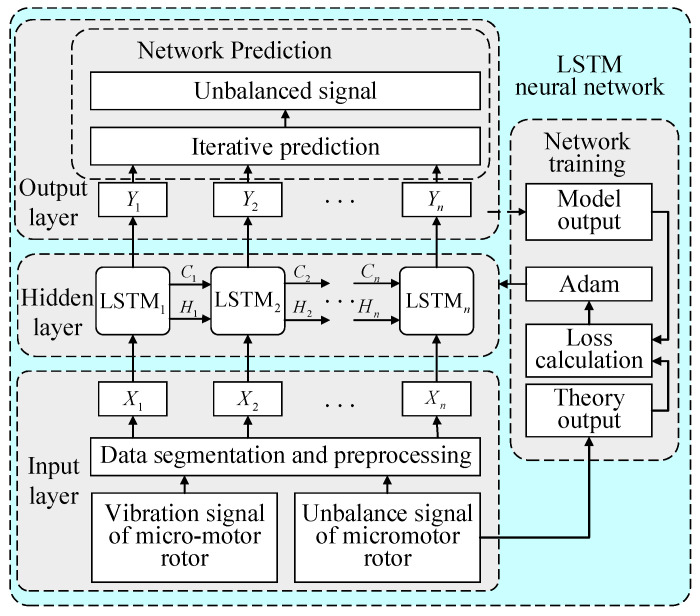
Training framework of LSTM network.

The LSTM neural network needs to be trained before use, and its network training framework is shown in Figure 5. For the motor armature vibration signal set *X* = {*X*_1_, *X*_2_, …, *X*_n_}, the corresponding theoretical output is the motor armature unbalance signal set *P* = {*P*_1_, *P*_2_, …, *P_n_*}. After inputting *X* into the hidden layer containing *n* isomorphic LSTM cells, the output is represented as *Y* = {*Y*_1_, *Y*_2_, …, *Y_n_*}.

In Figure 5, *C_n_*_−1_ and *P_n_*_−1_ are the state and output of the previous LSTM cell, respectively, and LSTM_n_ denotes the calculation method mentioned above in Equations (7)–(15). Minimizing the loss function is set as the optimization objective [35], the gradient-based optimization algorithm is applied to update the weights [36,37,38], and the Adam optimization algorithm [39] is chosen in this paper, with simple implementation and little memory required, resulting in better overall performance in practical applications. The trained LSTM neural network is used to learn and processing the unbalanced signal of the motor armature.

ZPF uses bandpass filtering to extract the desired amplitude of the motor armature unbalance signal. In this paper, the window function method is chosen to design the filter, and the Hanning window is selected [40], which has a large side flap attenuation and a strong attenuation capability for the interference signal that can effectively reduce the spectrum leakage during the spectrum analysis [41].

Signal fitting was performed by least squares fitting [42] to extract the motor armature unbalance signal amplitude information predicted by the LSTM neural network and the motor armature unbalance signal phase information analyzed by ZPF. The two are combined with the rotational speed information to obtain the final predicted motor armature unbalance signal. The method combines the advantages of LSTM neural network prediction and ZPF, with a better filtering effect and more accurate filtered signal.

In the proposed method, the unbalanced signal extraction is the most important for dynamic balancing of motor armature, which is the focus of this paper.

### 4.2. Evaluation Metrics

To evaluate the performance, five evaluation metrics are used. The amplitude determination coefficient $r_{V}^{2}$ and the root-mean-square error of amplitude $RMSE_{V}$ are two evaluation indicators that reflect the accuracy of amplitude prediction extraction. They are expressed as follows:

(21) $$r_{V}^{2}=1-\sum_{i=1}^{N}{\left(V_{i}-{\hat{V}}_{i}\right)}^{2}/\sum_{i=1}^{N}{\left(V_{i}-{\bar{V}}_{i}\right)}^{2},$$

(22) $$RMSE_{v}=\sqrt{\sum_{i=1}^{N}{\left({\hat{V}}_{i}-V_{i}\right)}^{2}/N},$$

where $V_{i}$ is the actual unbalance signal amplitude, ${\bar{V}}_{i}$ is its average value, ${\hat{V}}_{i}$ is the predicted unbalance signal amplitude, and N is the number of testing samples.

The phase determination coefficient $r_{\varphi }^{2}$, the root-mean-square error of phase $RMSE_{\varphi }$, and the mean absolute error of phase $MAE_{\varphi }$ are three evaluation indicators that reflect the accuracy of phase prediction extraction. They are expressed as follows:

(23) $$r_{\varphi }^{2}=1-\sum_{i=1}^{N}{\left(\varphi_{i}-{\hat{\varphi }}_{i}\right)}^{2}/\sum_{i=1}^{N}{\left(\varphi_{i}-{\bar{\varphi }}_{i}\right)}^{2},$$

(24) $$RMSE_{\varphi }=\sqrt{\sum_{i=1}^{N}{\left({\hat{\varphi }}_{i}-\varphi_{i}\right)}^{2}/N},$$

(25) $$MAE_{\varphi }=\sum_{i=1}^{N}\left|{\hat{\varphi }}_{i}-\varphi_{i}\right|/N,$$

where $\varphi_{i}$ is the actual unbalance signal phase, ${\bar{\varphi }}_{i}$ is its average value, and ${\hat{\varphi }}_{i}$ is the predicted unbalance signal phase.

The determination coefficient reflects the degree of fit of the predicted and actual values, the root-mean-square error reflects the deviation between the predicted and actual values, and the mean absolute error accurately reflects the magnitude of the actual prediction error.

## 5. Case Studies

### 5.1. Simulated Signal

#### 5.1.1. Data

**Figure 6:**
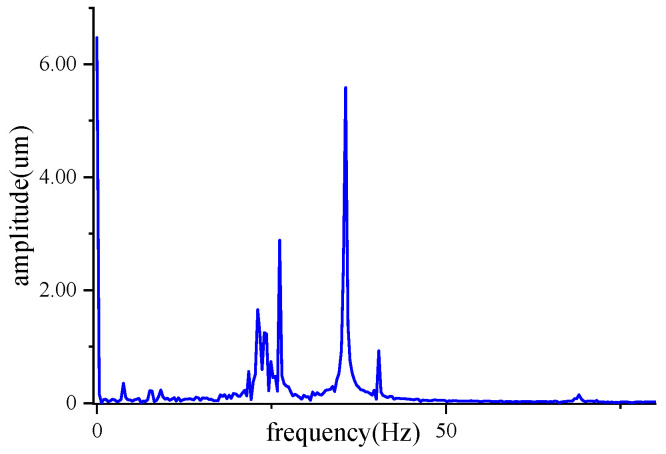
Frequency spectrum of armature vibration signal.

The frequency spectrum of the vibration signal of a motor armature when it is dynamically balanced in a laboratory environment is shown in Figure 6. It mainly consists of the unbalance signal generated by the unbalanced mass at the operating frequency, the DC component, the vibration signal of the inherent frequency of the support frame, the operating frequency signal of the equipment fan, and other superimposed sinusoidal signals. Since the existing signal processing methods cannot get the exact unbalance signal of the armature, this paper uses simulation data training to minimize the error and ensure the accuracy of the data, before using the actual armature for verification.

The motor armature vibration signal is modeled on the basis of the frequency spectrum analysis of the motor armature vibration signal, and Gaussian noise is added to mimic the factory noise environment, as expressed below.

(26) $$\left\{ \begin{array}{l} Y_{0}=A_{0}\sin\left(\omega_{0}x+c_{0}\right)\\ Y_{i}=A_{i}\sin\left(\omega_{i}x+c_{i}\right) \end{array}\right.,$$

(27) $$Y=Y_{0}+\sum_{i=1}^{i}Y_{i}+\mu ,$$

where *Y*_0_ is the armature unbalance signal, *Y* is the vibration signal, *Y_i_* is the inherent frequency vibration or industrial frequency vibration of each equipment of the system, *A* is the signal amplitude, *ω* is the signal frequency, *c* is the signal phase, and *μ* is the Gaussian noise.

**Figure 7:**
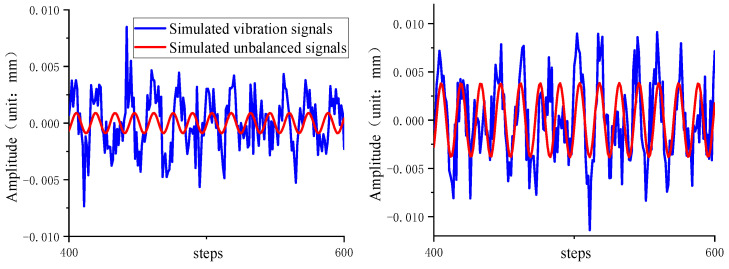
Simulated signal with local enlargement.

According to the vibration amplitude range of the motor armature unbalance signal provided by the factory, the datasets were constructed by selecting data with random phase in the amplitude range of 0.005–0.09 mm at equal intervals. A total of 500 sets of data were selected at equal intervals as the test set, and the remaining 4500 sets of data were used as the training set. Figure 7 shows the local signal plots for two of the randomly selected data sets.

#### 5.1.2. Simulation Verification

The signal processing part of the proposed method was simulated and verified using an Intel(R) i7 8750H CPU on Windows 10 OS. The programming and the deep learning platforms were both Matlab. In order to verify the feasibility of the proposed model, it was compared with five methods. The amplitude and phase results are shown below.

**Figure 8:**
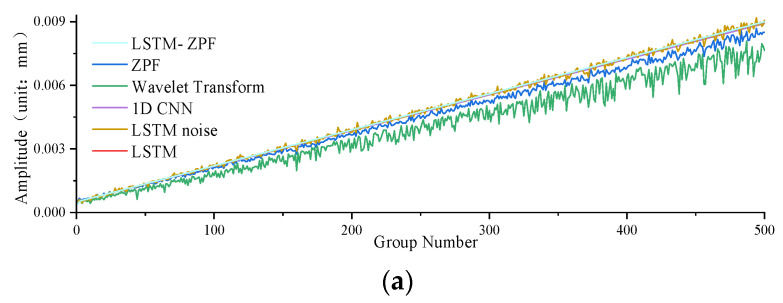

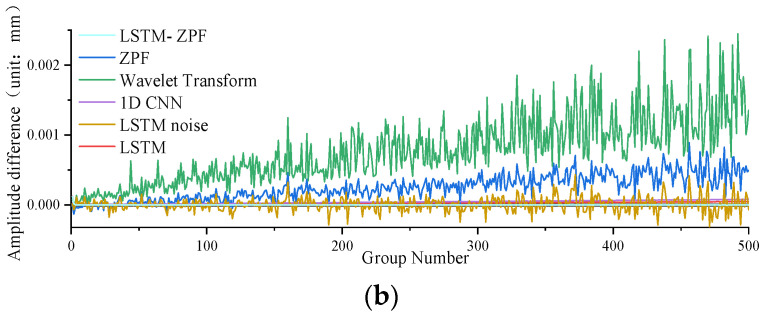
Comparison of amplitude accuracy: (**a**) comparison of predicted amplitude and ideal amplitude of different methods; (**b**) difference between predicted amplitude and ideal amplitude of different methods.

**Figure 9:**
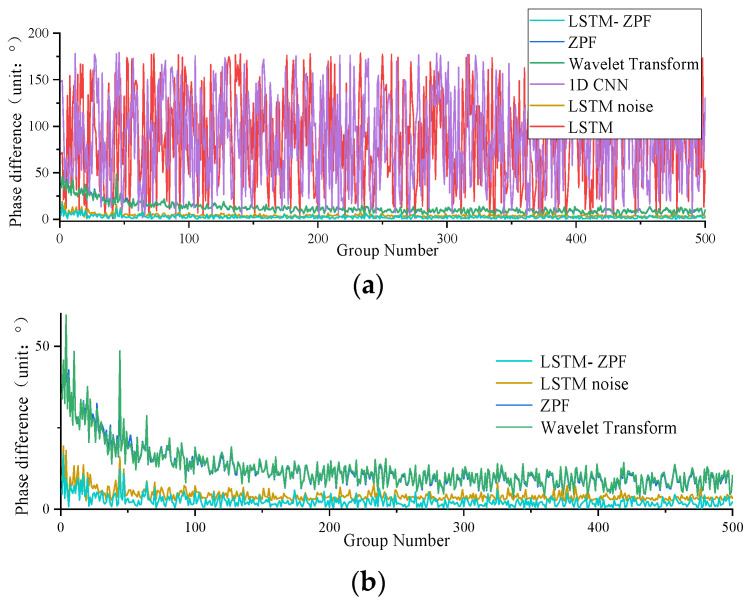
Comparison of phase accuracy: (**a**) phase difference of the predicted signal using different methods; (**b**) phase difference of the signal predicted using the method of effective phase extraction.

**Figure 10:**
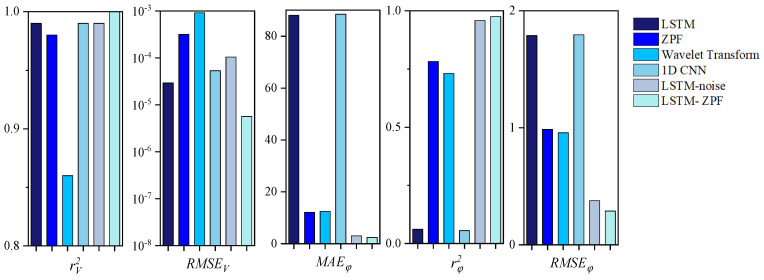
Comparison of evaluation indicators.

Figure 8, Figure 9 and Figure 10 and Table 1 show a comparison of the extraction accuracy of the unbalance signal of the motor armature using different methods. It can be seen that the proposed method outperformed other methods in terms of amplitude and phase extraction of the motor armature vibration signal. ZPF and wavelet transform are two existing methods. The amplitude extraction accuracy of ZPF is not bad, but it is lower than that of the proposed method. Due to the existence of near-frequency interference, ZPF cannot really achieve zero-phase extraction of the motor armature unbalance signal, which leads to a phase shift of about 12°. Meanwhile, the signal amplitude of ZPF has attenuation. The analysis of ZPF was applied to wavelet transform. In addition to the conventional methods, three neural network models were used for comparison in this paper. The first, LSTM, can achieve accurate extraction of the amplitude, with the same obvious weakness that phase information cannot be extracted. The second is the 1D convolutional neural networks (1D CNN) [43,44,45], which has a lower amplitude prediction extraction than the proposed method and cannot extraction the phase. The third is LSTM-noise, which is another model proposed by the authors. This method predicts the noise disturbance signal through the LSTM network and then implements backstepping to achieve the motor armature unbalance signal; this method enables better extraction for both amplitude and phase, but the accuracy is lower while the operation time consumed is much higher than the proposed method.

#### 5.1.3. Anti-Noise Performance Analysis

In the actual plant environment, the noise disturbance is always changing in real time. In order to verify the stability of the proposed method, this paper changed the signal-to-noise ratio (SNR) in the vibration signal data, and then investigated the effect of the proposed method on the extraction accuracy of the motor vibration signal extraction for different SNRs.

Table 2 shows the amplitude and phase accuracy of the vibration signals predicted using the LSTM-ZPF method for motor armature unbalance signals at different SNRs (10, 20, 30, and 40). The following can be concluded:

1. The amplitude fitting accuracy of the training set corresponding to the five SNRs is basically the same, and the accuracy is high;
2. The phase fitting accuracy of the training set corresponding to the four SNRs is also basically the same. For a higher noise, there is lower phase accuracy, but the phase error is within an acceptable range, and all results exceeded those of the other filtering methods mentioned above;
3. This method can effectively extract the motor armature unbalance signal at different SNRs, which makes the armature unbalanced mass calculation more accurate. It has good stability.

**Table 2:** Comparison of the results of LSTM-ZPF methods with different SNRs.

SNR	Amplitude		Phase (°)		
	$r_{V}^{2}$	$RMSE_{V}$	$MAE_{\varphi }$	$r_{\varphi }^{2}$	$RMSE_{\varphi }$
40	0.9999	2.9288 × 10^−5^	1.8626	0.9821	0.2472
30	0.9999	2.7436 × 10^−5^	1.8604	0.9822	0.2466
20	0.9999	2.8445 × 10^−5^	1.8567	0.9817	0.2496
10	0.9999	2.1446 × 10^−5^	2.0650	0.9796	0.2636

### 5.2. Experimental Verification

**Figure 11:**
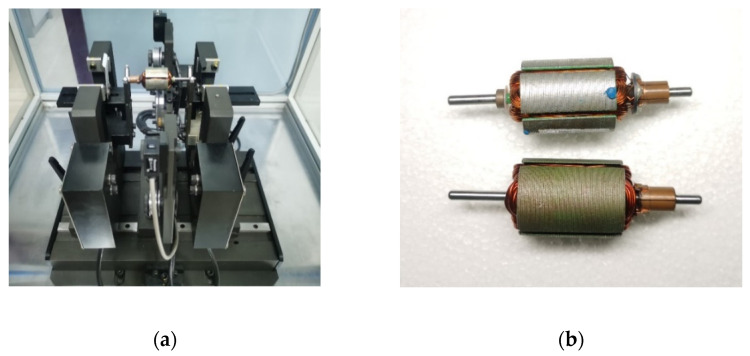
Dynamic balancing experimental equipment: (**a**) dynamic balancing experimental platform; (**b**) motor armature after dynamic balancing.

The dynamic balancing experimental platform is shown in Figure 11a. It was mainly composed of support brackets, a stepper motor, a pulley, sensors, and a data processing system. Its unbalanced signal extraction principle is shown in Figure 1. Two support brackets were located on both sides of the platform, the pulley was connected to the stepper motor by a belt, the two displacement sensors faced each of the two support brackets, and the photoelectric sensor was located between the support brackets. When extracting an unbalance signal, the rotor is placed on the support brackets. Its underside is contacted with the belt, and the stepper motor powers the rotation. The displacement sensors measure the vibration of the support brackets and, thus, indirectly measure the vibration of the two correction planes, while the photoelectric sensor measures the phase of the rotor. The data processing system calculates the unbalanced mass and phase.

Since the ZPF performed best in the previous experiments among methods existing in the literature, the proposed method and the ZPF were used to extract the amplitude and phase of the unbalance signal of the armature. ZPF was applied to the chip in the dynamic balancing experimental platform. Both methods were used to calculate the armature unbalanced mass and phase of the same armature, and then the mass was weighted separately. Then, a frequency spectrum analysis of mass weighted on the armature was established to determine the accuracy of the armature dynamic balancing.

As shown in Figure 11b, a five-slot motor armature and a three-slot motor armature were selected. After measuring the mass-unweighted, mass left-weighted, and mass right-weighted vibration signals, the armature unbalanced mass and phase were calculated using the proposed method and ZPF. The calculation results are shown in Table 3.

On the basis of the data calculated in Table 3, the mass was added in the opposite direction of the armature. Then, the proposed method was used to calculate the unbalance signal amplitude of the motor armature after dynamic balancing by both methods. The results are shown in Table 4.

**Figure 12:**
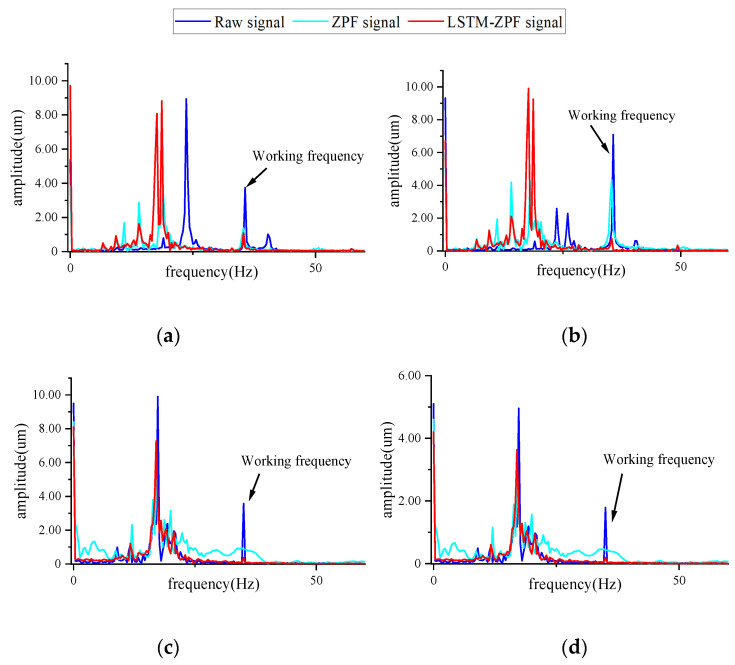
Frequency spectrum of vibration signal of armature correction planes: (**a**) left correction plane of armature No. 1; (**b**) right correction plane of armature No. 1; (**c**) left correction plane of armature No. 2; (**d**) right correction plane of armature No. 2.

As can be seen from Table 4, the amplitude of armature vibration after dynamic balancing using the proposed method was smaller than that of the ZPF. Since the filtering may cause signal amplitude loss and phase shift, the armature vibration signal spectrum after the test weight of different methods was directly analyzed at the same time. The frequency spectrum of the armature vibration signal after dynamic balancing is shown in Figure 12. The vibration response of the armature at working frequency was significantly reduced after dynamic balancing. The working frequency amplitude of the armature after balancing with the proposed method was far smaller than that of the ZPF. This proved that the proposed method is more effective in dynamic balancing. It can be seen that the amplitude of other frequencies also changed to varying degrees. This is because, during the dynamic balance test, the centrifugal force generated by the unbalanced mass of the motor armature is the main source of force on the dynamic balance test system. The mass change in motor armature leads to a change in the force on the dynamic balance test system, which affects the vibration at different frequencies.

## 6. Conclusions

A dynamic balancing method of the motor armature based on signal processing using an LSTM integrated with ZPF was proposed. The effectiveness of the proposed method was verified through dynamic balancing experiments on the motor armature. The proposed method could accurately obtain the unbalanced mass and phase of the armature. Both fidelity and denoising were considered in the proposed method during signal processing, and the problem of amplitude loss in extracting the phase was solved. The phase of the signal could be better extracted with almost no phase shift, and the mean absolute error of the phase was 2.4°. The amplitude obtained using the proposed method was less decayed compared with the ideal spectrum curve, and the determination coefficient of the amplitude was 0.9999. The interference of noise could be eliminated using the proposed method, while the amplitude and phase extraction accuracy of the unbalanced signal could be obtained even in noisy environments. Different types of motor armature dynamic balancing are suitable for the proposed method.

## Figures and Tables

**Table 1:** Comparison of evaluation indicators.

Methods	Amplitude		Phase (°)		
	$r_{V}^{2}$	$RMSE_{V}$	$MAE_{\varphi }$	$r_{\varphi }^{2}$	$RMSE_{\varphi }$
LSTM	0.9999	2.9280 × 10^−5^	88.0613	0.0611	1.7884
ZPF	0.9836	3.1447 × 10^−4^	12.1589	0.7821	0.9868
Wavelet Transform	0.8613	9.1371 × 10^−4^	12.4614	0.7308	0.9577
1D CNN	0.995	5.3350 × 10^−5^	88.4542	0.0552	1.7941
LSTM-noise	0.9982	1.0440 × 10^−4^	3.0401	0.9583	0.3767
LSTM-ZPF	0.9999	5.6705 × 10^−6^	2.4069	0.9756	0.2885

**Table 3:** Calculated results of motor armature unevenness.

No.	Methods	Left Unbalanced Mass (mg)	Left Phase (°)	Right Unbalanced Mass (mg)	Right Phase (°)
1	LSTM-ZPF	72.4	3.0	77.5	146.0
	Hardware filtering (ZPF)	76.2	6.3	75.3	139.2
2	LSTM-ZPF	64.7	243.8	27.4	224.5
	Hardware filtering (ZPF)	70.5	210.1	30.6	212.6

**Table 4:** Correction plane amplitude after dynamic balancing of motor armature.

Methods	Left Amplitude (μm)		Right Amplitude (μm)	
No.	1	2	1	2
LSTM-ZPF	1.13	0.75	0.89	0.66
Hardware filtering (ZPF)	1.34	1.09	4.71	0.98

## Data Availability

Not applicable.
